# Go go gadget glycoprotein!: HSV-1 draws on its sizeable glycoprotein tool kit to customize its diverse entry routes

**DOI:** 10.1371/journal.ppat.1007660

**Published:** 2019-05-09

**Authors:** Adam T. Hilterbrand, Ekaterina E. Heldwein

**Affiliations:** Department of Molecular Biology and Microbiology, Tufts University School of Medicine, Boston, Massachusetts, United States of America; University of Michigan Medical School, UNITED STATES

## Introduction: Viruses deploy diverse entry strategies

All viruses must enter cells to replicate [1]. Entry is thus the first hurdle a virus must overcome for a successful infection. Given the astonishing diversity of viruses that infect mammals alone—hundreds of thousands of different viruses according to some estimates [2]—it is unsurprising that their entry routes into cells are just as diverse. A major structural distinction that dictates the entry mode is the presence or absence of a lipid bilayer surrounding the viral nucleocapsid, which defines a virus as enveloped or nonenveloped, respectively. Nonenveloped viruses enter cells by endocytosis and subsequently penetrate the endosomal membrane by a variety of mechanisms including pore formation and endosomal fragmentation due to disruptive changes in membrane curvature [3], whereas all enveloped viruses must fuse their envelope with a host cell membrane: either the plasma membrane or the membrane of the endosomal vesicle following cellular uptake [4].

Regardless of the entry route, all viruses initially attach to the surface of the host cell by binding a cellular receptor. After attachment, enveloped viruses must employ fusogens—specialized viral surface glycoproteins that mediate the merger of the viral and host membranes by bringing them together as they undergo large, energetically favorable conformational changes. To do this, a spring-loaded fusogen must be triggered once the virus arrives at the right cell and/or the right intracellular compartment (such as an endosome, for example), either by binding a receptor (or a coreceptor) or by sensing the acidic pH of the endosome [4]. In many enveloped viruses, the receptor-binding and the fusogenic functions are mediated by different domains of a single glycoprotein. For example, the human immunodeficiency virus (HIV) envelope protein, Env, the sole glycoprotein encoded by HIV, binds the cellular glycoprotein cluster of differentiation 4 (CD4) and a coreceptor, C-X-C chemokine receptor 4 (CXCR4) or C-C chemokine receptor 5 (CCR5), on the surface of CD4^+^ T cells and also serves as the fusogen [5]. The influenza virus glycoprotein hemagglutinin binds an attachment receptor, sialic acid, and undergoes low-pH-triggered fusogenic conformational changes upon endocytosis [6]. In some cases, for example, in paramyxoviruses, the receptor-binding and the fusogenic functions are mediated by separate glycoproteins, and the fusogen receives the triggering signal from the receptor-binding viral protein [7]. Most enveloped viruses thus contain multiple copies of only one or two glycoproteins, which mediate viral attachment and entry into target cells [5–15].

Yet, entry of herpesviruses—large enveloped viruses that infect a wide variety of cells—is more complex, as it requires multiple viral glycoproteins (typically, at least three) and diverse host receptors [16]. Moreover, the coordinated activity of these multiple viral glycoproteins permits entry into different cell types by different routes. Whereas in some herpesviruses, such as human cytomegalovirus (HCMV) or Epstein–Barr virus (EBV), the use of particular entry routes correlates with the involvement of specific viral glycoprotein complexes [17, 18], in other herpesviruses, notably, herpes simplex virus type 1 (HSV-1), the picture is less clear [19]. Nonetheless, the entry mechanisms of all herpesviruses into a given cell, and particularly, the selection of the entry route, are complex and incompletely understood.

The HSV-1 replication cycle in humans necessitates the infection of different cell types, chiefly, epithelial and neuronal cells. Although it is known that HSV-1 enters these cells by different mechanisms—endocytosis (epithelial cells) and fusion at the plasma membrane (neurons) [20, 21]—knowledge regarding HSV-1 glycoprotein involvement in the entry route–selection process is minimal. This raises the following question: How does HSV-1 select a particular route to enter different cell types? Although the answer remains elusive, this Pearl will summarize the current understanding of HSV-1 entry strategies and the players involved.

## The HSV-1 envelope contains over a dozen proteins, but only four are required for entry

**Fig 1:**
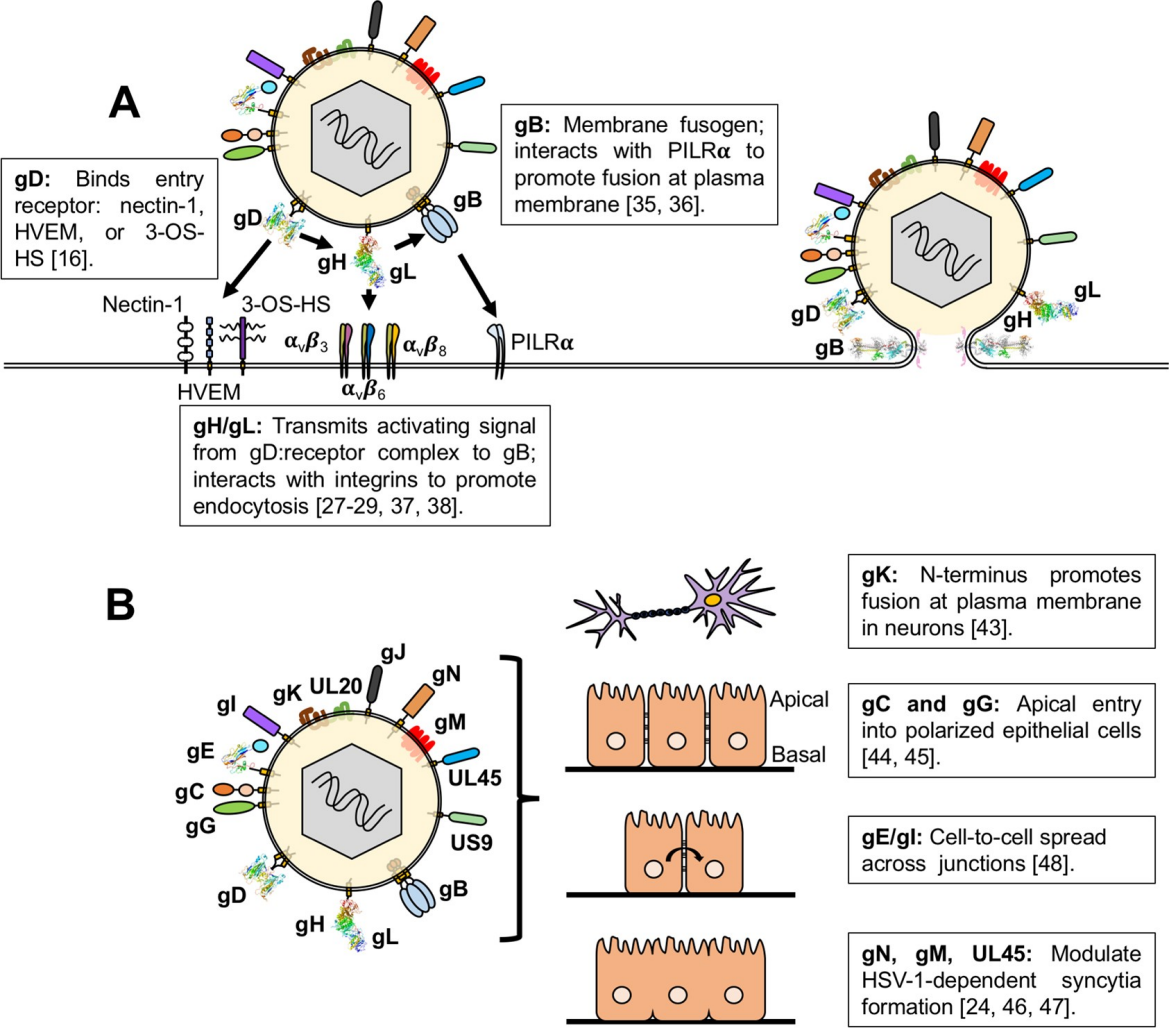
HSV-1 envelope proteins and their roles in entry and membrane fusion. (A) HSV-1 entry into cells requires the coordinated efforts of the receptor-binding glycoprotein gD (RCSB PDB: 2C36), the heterodimer gH/gL (RCSB PDB: 3M1C), and the fusogen gB (RCSB PDB: 5V2S). For gB, only the structure of its postfusion conformation is known, so the prefusion conformation of HSV-1 gB is depicted schematically. Interactions of these essential proteins with cellular coreceptors can influence the entry of HSV-1 into a cell. (B) HSV-1 contains 15 proteins within its lipid envelope, 12 glycosylated (gB, gC, gD, gE [RCSB PDB: 2GIY], gG, gH, gI, gJ, gK, gL, gM, gN) and three unglycosylated (UL20, UL45, US9). The roles of the 11 “nonessential” envelope proteins, with respect to entry route selection, are minimally understood. Glycoproteins gC and gG promote entry at the apical side of polarized epithelial cells. The glycoprotein gK promotes entry into neurons by fusion at the plasma membrane. Other envelope proteins (gE, gI, gM, gN, and UL45) have roles in cell-to-cell spread and membrane fusion but have not yet been assigned any roles in entry. 3-OS-HS, 3-O-sulfated-heparan sulfate; HSV-1, herpes simplex virus type 1; HVEM, herpes virus entry mediator; PDB, Protein Data Bank; PILRα, paired immunoglobulin-like type 2 receptor alpha; RCSB, Research Collaboratory for Structural Bioinformatics.

HSV-1 contains 15 viral proteins in its lipid envelope, 12 glycosylated and three unglycosylated (Fig 1B) [19]. Four of these glycosylated proteins—gD, gH, gL, and gB—are essential for entry into target cells in tissue culture and in animal models (Fig 1A) [22, 23], whereas the other 11 proteins are typically referred to as “nonessential” with regard to entry because their deletions have mild phenotypes, if any, in cell culture [24–26].

HSV-1 entry into any cell requires the coordinated efforts of gD, gH, gL, and gB. It is now known that in HSV-1 the receptor-binding and the fusogenic functions are distributed among these four glycoproteins [23]. First, gB (along with another glycoprotein, gC) mediates viral attachment to cell-surface heparan sulfate proteoglycans [22]. Next, HSV-1 uses its receptor-binding glycoprotein, gD, to engage one of its three entry receptors: nectin-1, herpes virus entry mediator (HVEM), or 3-O-sulfated-heparan sulfate (3-OS-HS) (Fig 1A) [16]. Binding to its receptor triggers a conformational change within gD that causes it to bind the heterodimer gH/gL [27–29]. This event, in turn, activates gB, the fusogen that mediates the merger of the HSV-1 lipid envelope with the cellular membrane [30]. gD, gH, gL, and gB are not only essential for HSV-1 entry but are also sufficient for entry of heterologous viral particles pseudotyped with these four glycoproteins [31]. Moreover, gD, gH, gL, and gB can also mediate cell–cell fusion of uninfected, receptor-bearing cells expressing these four glycoproteins [23]. Collectively, these data suggest that gD, gH, gL, and gB represent the core receptor-binding/fusogenic machinery of HSV-1. Given that gD, gH, gL, gB, and a gD receptor are required for entry regardless of cell type or entry route, how does HSV-1 choose which route to take?

In some cases, entry route depends on cell type–specific interactions of gD, gH/gL, or gB with host receptors and the HSV-1 strain [32–34]. For example, HSV-1 enters nectin-1-bearing Chinese hamster ovary (CHO) cells by endocytosis, but overexpression of the cellular protein paired immunoglobulin-like type 2 receptor alpha (PILRα)—which binds gB (Fig 1A)—switches the entry route to fusion at the plasma membrane [35, 36]. Similarly, HSV-1 entry into nectin-1-bearing CHO and J cells (a baby hamster kidney [BHK] cell derivative) by endocytosis requires both dynamin and cholesterol only when integrins α_v_β_3_, α_v_β_6_, or α_v_β_8_, which bind gH/gL (Fig 1A), are present [37, 38]. These studies indicate that cellular interactions with gD, gH/gL, or gB can direct the entry route or change the cellular requirements for HSV-1 entry.

## “Nonessential” HSV-1 envelope proteins influence HSV-1 entry pathways and fusion-dependent processes

Given these observations, one may think that gD, gH, gL, gB, and a cellular receptor for gD are all that HSV-1 needs to enter any target cell. However, the presence of 11 additional envelope proteins (eight glycosylated and three unglycosylated) suggests that they serve important roles during infection. This notion is supported by the observation that even highly passaged tissue culture–adapted strains of HSV-1 retain these envelope protein genes [39], in contrast to herpesviruses such as HCMV, which rapidly loses certain glycoprotein genes or their portions during passaging in tissue culture [40]. Even though these proteins are not required for HSV-1 entry [24–26], their interactions with cellular factors, gD, gH, gL, gB, or some combination thereof, could influence the pathway HSV-1 takes into a cell.

Indeed, several studies have revealed context-dependent contributions of the “nonessential” envelope proteins (Fig 1B). For example, initial studies of glycoprotein gK demonstrated that gK was dispensable for HSV-1 entry even though its deletion reduced entry efficiency [41], consistent with the “nonessential” designation. However, later work revealed that gK enabled HSV-1 to enter neuronal cells by fusion at the plasma membrane [42]. When the amino terminus of gK was deleted, HSV-1 switched the entry route to clathrin- and dynamin-dependent endocytosis [43], suggesting that the amino terminus of gK is essential for HSV-1 entry by fusion at the plasma membrane, in the context of neuronal cells (Fig 1B). Similar to gK, the glycoproteins gG and gC are dispensable for HSV-1 entry into non-polarized epithelial cells [25, 26]. However, under culture conditions that produce polarized epithelial cells, both gC and gG promote HSV-1 entry at the apical side of these cells, whereas entry at the basal side does not require either protein (Fig 1B) [44, 45]. This suggests that gC and gG promote HSV-1 entry either by binding some necessary apically localized host factor or by overcoming a host restriction factor that normally prevents HSV-1 entry at the apical side of a polarized epithelial cell. These studies indicate that the so-called “nonessential” glycoproteins can serve to facilitate the selection of HSV-1 entry points. In other words, these proteins may guide HSV-1 into the most efficient “entry lane”; without them, the virus would still enter cells, albeit less efficiently. In this manner, these “nonessential” proteins may serve to increase HSV-1 fitness. Although the rest of the “nonessential” envelope proteins have not yet been ascribed clear roles in HSV-1 entry pathways, they do influence membrane fusion and cell-to-cell spread of HSV-1 (Fig 1B), processes that are both important for HSV-1 pathogenesis. For example, UL45, one of the three unglycosylated envelope proteins, and the glycoproteins gM and gN promote the formation of syncytia [25, 46, 47]. Likewise, the glycoproteins gE and gI, although not essential for entry, promote cell-to-cell spread [48]. It is tempting to speculate that, given their important roles in membrane fusion and cell-to-cell spread, these five envelope proteins may also contribute to HSV-1 entry mechanisms in certain cell-specific contexts.

## Conclusion

HSV-1 entry is an enigmatic process, and despite much progress over the past half century, many questions about its entry process remain. How does HSV-1 choose its pathway into a cell? What cellular factors and viral proteins govern this choice? What are the molecular mechanisms surrounding the different HSV-1 entry pathways? Currently, we know that host factor–specific interactions with HSV-1 envelope proteins seem to dictate the way HSV-1 enters a given cell. Although cell-specific interactions of the essential entry proteins (gD, gH/gL, and gB) with host factors can alter HSV-1 entry modes, “nonessential” envelope proteins have been shown to make important contributions to HSV-1 entry in specific circumstances. Just as the cartoon character Inspector Gadget has the ability to call upon any number of tools to help him defeat criminals, HSV-1 may be able to call upon a large number of envelope glycoproteins and unglycosylated proteins to help it enter receptor-bearing cells in the most efficient way. Efforts characterizing the roles of gK, gC, and gG have lent credence to the hypothesis that envelope proteins other than gD, gH/gL, and gB can influence entry pathways and therefore warrant further investigation. Our current knowledge of the roles of the essential HSV-1 glycoproteins gD, gH/gL, and gB in HSV-1 entry has greatly benefited from a multidisciplinary approach combining structural biology, cell biology, biochemistry, and genetics. These approaches are now called for in investigating the roles of the “nonessential” envelope proteins in entry. A detailed understanding of HSV-1 entry strategies will inform the development of efficacious inhibitors and vaccines.
